# Inhibition of *Clostridium perfringens* Spore Germination by the Synergistic Effects of the Natural Products Chitosan and Nisin

**DOI:** 10.3390/microorganisms13092116

**Published:** 2025-09-10

**Authors:** Rabiaa S. Alhabeeb, Roua Almatrafi, Saeed S. Banawas, Maryam Alnoman, Mahfuzur R. Sarker

**Affiliations:** 1 Department of Biomedical Sciences, Carlson College of Veterinary Medicine, Oregon State University, Corvallis, OR 97331, USA; rsalhabeeb@moh.gov.sa; 2 King Abdul Aziz Hospital, Makkah, Makkah 4222, Saudi Arabia; 3 Department of Biology, College of Science, University of Bisha, Bisha 61922, Saudi Arabia; ralmatrafi@ub.edu.sa; 4 Department of Medical Laboratories, College of Applied Sciences, Majmaah University, Al-Majmaah 11952, Saudi Arabia; 5 Department of Biology, College of Science Yanbu, Taibah University, Al-Madinah 46423, Saudi Arabia; mnaaman@taibahu.edu.sa; 6 Department of Microbiology, College of Science, Oregon State University, Corvallis, OR 97331, USA

**Keywords:** *Clostridium perfringens*, spores, germination, chitosan, nisin, inhibition

## Abstract

*Clostridium perfringens* is a spore-forming bacterium that causes food poisoning. Given the high heat resistance of its spores, natural antimicrobial agents are considered as alternatives to thermal processing strategies to inactivate or eliminate such spores from food products. A high chitosan concentration (0.2%) can effectively inhibit the growth of *C. perfringens* spores in cooked chicken meat, whereas nisin cannot (even at concentrations four times higher than those permitted: 250 μM). However, nisin is an effective inhibitor when in combination with other preservatives. Therefore, we evaluated the inhibitory effects of a chitosan–nisin combination on the germination, outgrowth, and vegetative growth of *C. perfringens* spores in laboratory medium and chicken meat. Among many tested concentration combinations, a 0.025% chitosan and 0.075% nisin mixture was found to be the most effective for inhibiting spore germination and outgrowth in laboratory medium. Furthermore, a mixture of chitosan–nisin, at 0.025% each, blocked the vegetative growth of *C. perfringens* spores. However, four-times higher concentrations of chitosan–nisin (0.1% each) were required to effectively inhibit *C. perfringens* spore germination in chicken meat. Collectively, our results suggest that the combination of chitosan and nisin can be considered as an alternative approach to control *C. perfringens* spore germination in meat products.

## 1. Introduction

*Clostridium perfringens* is an anaerobic spore-forming organism, commonly found in soils, that can cause serious diseases in humans and animals [1]. The virulence of *C. perfringens* is largely attributable to its ability to produce at least 20 different toxins [2,3]. Among the seven types of *C. perfringens* (A–G), the enterotoxin-producing type F is the most common cause of human foodborne and non-foodborne illnesses and outbreaks worldwide [2]. Additionally, *C. perfringens* type F isolates produce metabolically dormant spores that can survive the preservation approaches commonly used to control spoilage and pathogenic microorganisms in food products [4]. These viable spores germinate, outgrow, and multiply to a great extent in food products, causing disease after the consumption of contaminated foods [5].

Mild heat treatments (55–75 °C) can be very effective in eliminating *C. perfringens* vegetative cells. In contrast, spores are highly resistant, and heat treatments may even activate dormant spores, allowing them to germinate and outgrow to hazardous levels thereafter [6]. Consequently, one of the main goals of the food industry is to establish new bacterial spore inactivation strategies, as an alternative to heat processing. Alternative technologies include the use of natural antimicrobial agents to ensure food protection, consistency, and shelf life by preventing microbial spoilage and contamination by food-borne pathogens [7,8]. Nisin, a 34-amino acid polypeptide produced by *Lactococcus lactis* subsp. *lactis* is a promising option because of its antimicrobial properties against foodborne pathogenic bacteria, such as *Listeria monocytogenes* and many other Gram-positive food spoilage microorganisms [8,9,10]. Nisin has been successfully used as a food preservative in different types of foods, including dairy and meat products, either alone or in combination with other preservatives or physical treatments [11,12].

Another interesting compound is chitosan, which has antimicrobial activity against many Gram-positive and Gram-negative bacteria as well as fungi [13,14,15]. Chitosan is a natural carbohydrate polymer that results from an incomplete deacetylation of chitin; its antibacterial activity depends on its molecular weight, degree of deacetylation, physical state, and pH [16]. Several reports have suggested that commercially available shrimp-derived chitosan can be used as a food preservative and an active packaging material [16,17,18].

Previous studies in our laboratory demonstrated the inhibitory effects of nisin and chitosan on *C. perfringens* type F isolates [19]. Under laboratory conditions, these antimicrobial agents slightly inhibited spore germination and significantly inhibited spore outgrowth and vegetative growth of *C. perfringens* isolates. However, in meat experiments, only chitosan effectively inhibited the germination of *C. perfringens* spores: a dose of 0.2% chitosan resulted in ~3 log colony-forming units (CFU)/g reduction in *C. perfringens* spore growth in cooked chicken meat [13]. In contrast, even at a concentration of 250 μM, which is four times higher than that permitted by the authorities, nisin could not inhibit *C. perfringens* spore germination in cooked chicken meat [19]. Therefore, it is necessary to develop new antimicrobials combining chitosan with other compounds at low concentrations to inhibit *C. perfringens* spore germination in cooked chicken meat. Recent studies have shown that nisin is effective when combined with other preservatives [11]. In this study, we aimed to evaluate the inhibitory effects of nisin and chitosan combinations on the germination of *C. perfringens* spores. Our results showed synergistic effects between chitosan and nisin in inhibiting *C. perfringens* spore germination in laboratory medium and chicken meat.

## 2. Materials and Methods

### 2.1. Bacterial Strains and Growth Conditions

*C. perfringens* type F food-borne isolates SM101, NCTC10239, E13, and NCTC8239 were used in this study. The isolates were maintained as stock cultures in cooked meat medium (BD Difco, BD Diagnostic Systems, Sparks, MD, USA) and stored at −20 °C.

### 2.2. Spore Preparation and Purification

Spores from all the isolates (SM101, NCTC10239, NCTC8239, and E13) were prepared and purified as previously described [20,21]. Briefly, a 0.1–0.2 mL aliquot of a stock culture in cooked meat medium was inoculated into 10 mL fresh fluid thioglycolate medium (FTG; BD Diagnostic Systems, Sparks, MD, USA) and incubated overnight at 37 °C. A 0.4 mL aliquot of this overnight culture was inoculated into 10 mL of fresh FTG medium and incubated at 37 °C for 9–12 h. Subsequently, 0.4 mL of the FTG culture was transferred to 10 mL Duncan-Strong (DS) sporulation medium (1.5% protease peptone, 0.4% yeast extract, 0.1% sodium thioglycolate, 0.5% sodium phosphate dibasic [Na_2_HPO_4_; anhydrous], and 0.4% soluble starch) [22] and incubated at 37 °C for 18–24 h. Spore formation was confirmed by visualizing sporulating cells under a phase-contrast microscope (Leica MDLS; Leica Microsystems, Wetzlar, Germany). This DS culture was then used as an inoculum to prepare large amounts of spores by scaling up the aforementioned procedure in 600 mL of fresh DS medium. The spores were purified by repeated sonication (2–3 times, 10 s each) and washed with cold sterile distilled water until 99% of phase-bright spores were obtained, as determined by phase-contrast microscopy. The purified spores were adjusted to a standardized optical density (OD_600_ ≈ 6.0) in sterile distilled water using a SmartSpec™ 3000 spectrophotometer (Bio-Rad, Hercules, CA, USA) and stored at −20 °C until use, consistent with established practices in *C. perfringens* spore methodologies.

### 2.3. Preparation of Antimicrobial Solutions

Low-molecular-weight chitosan (50–190 kDa), 75–85% deacetylated, was purchased from Sigma-Aldrich (St. Louis, MO, USA). A chitosan stock solution (10 mg/mL) was prepared in 1% (*v*/*v*) acetic acid [23,24], filter sterilized (0.45 µm; Millipore, Bedford, MA, USA), and used within 1 month of storage at 4 °C. A 4.0 mM stock solution of nisin (consisting of 2.5% pure nisin, with an estimated potency of 10^6^ IU g^−1^; Sigma Aldrich) was prepared in 0.02 N HCl, filter sterilized (0.45 μm; Millipore), and used within 1 week of storage at 4 °C.

### 2.4. C. perfringens Spore Germination in the Presence of Antimicrobial Agents

Spore germination was performed as previously described [19]. Briefly, a purified spore suspension (OD_600_ ≈ 6.0) was heat-activated for 10 min at 80 °C, cooled in a water bath at 20–25 °C for 5 min, mixed (33 μL) with TGY broth (3% trypticase, 2% glucose, 1% yeast extract, and 0.1% L-cysteine) either alone or supplemented with different concentrations of antimicrobials to a total volume of 0.2 mL per well in a 96-well microtiter plate, and incubated at 37 °C. OD_600_ was measured every 10 min using a Synergy TMMX multi-mode microtiter plate reader (BioTek Instruments Inc., Winooski, VT, USA). The extent of germination was expressed as the percentage of decrease in OD_600_ relative to the initial OD_600_ value. An approximate 60% decrease in OD_600_ compared with the initial value indicates complete spore germination. Spore germination level was also confirmed using phase-contrast microscopy, as fully germinated spores change from fully bright to phase-dark spores. Germination inhibition was calculated as previously described [25] using the following formula:

Germination inhibition (%) = (OD_600_ decrease in treatment/OD_600_ decrease in control) × 100

All experiments were repeated at least three times with different spore preparations for each of the strains tested in this study.

### 2.5. C. perfringens Spore Outgrowth in the Presence of Antimicrobials

A spore suspension with an OD_600_ of ~ 6.0 was heat-activated for 10 min at 80 °C and then cooled in a water bath at room temperature for 5 min. Subsequently, 0.3 mL of the heat-activated spore suspension was inoculated into TGY broth (pH 6.0) alone (control) or supplemented with various concentrations of antimicrobials. After incubation at 37 °C, OD_600_ was measured at 30 min intervals for up to 180 min postinoculation. The outgrowth rate of the spores was defined as the percentage increase in OD_600_. In addition, the percentage of inhibition of spore outgrowth was calculated as previously described [25] using the following formula:

Spore outgrowth inhibition (%) = (OD_600_ increase in treatment/OD_600_ increase in control) × 100

The experiments were conducted in triplicate using different spore preparations. Both spores and vegetative cells were kept and manipulated under anaerobic conditions.

### 2.6. C. perfringens Vegetative Growth in the Presence of Antimicrobials

Stock cultures of *C. perfringens* in cooked meat medium were inoculated (0.1 mL) into FTG and incubated for 18–24 h at 37 °C, and 0.4 mL aliquots of these cultures were each transferred into 10 mL of TGY and incubated at 37 °C for 3 h. Subsequently, 0.4 mL of each culture was transferred into 10 mL TGY, either alone as a control or supplemented with various concentrations of antimicrobials. Vegetative growth was measured by counting CFU/mL from samples collected after incubation at 37 °C for 3, 6, and 9 h. Briefly, aliquots of the samples taken at different times were serially diluted in 25 mM Na_2_HPO_4_ buffer (pH 7.5), plated on brain heart infusion (BHI; BD Diagnostic Systems, Sparks, MD, USA) agar plates, and incubated at 37 °C for 18–24 h. Formed colonies were counted and used to calculate the bacterial concentrations (CFU/mL). All experiments were performed in triplicate, and the values are presented as the mean.

### 2.7. Growth of C. perfringens Spores in Chicken Meat in the Presence of Antimicrobials

The growth of *C. perfringens* spores in chicken meat supplemented with different concentrations of antimicrobials was measured following a standard procedure. Briefly, 10 g of ground chicken meat was placed into a UV-sterilized plastic bag (5.5″ W × 6″ L) (Seal-a-Meal vacuum storage bag, Sunbeam Products, Inc., Boca Raton, FL, USA), sealed, autoclaved, and stored at −20 °C until use. For each experiment, frozen chicken meat samples were thawed at room temperature, and appropriate amounts of antimicrobials together with 100 μL of a 4-strain spore cocktail were then added. After resealing the bags, spores and antimicrobials were mixed with the meat by manual massaging for 1 min. Four-strain spore cocktails of food-poisoning (FP) bacterial isolates (SM101, NCTC10239, NCTC8239, and E13) were prepared by combining equal numbers of spores from each strain to a total of 10^8^ spores/mL. Chicken meat samples were cooked for 20 min at 80 °C and then cooled for 10 min in a water bath at room temperature. Sterile cooked chicken meat was used as a negative control for each replicate to ensure that the meat samples were free of naturally occurring bacteria. In each treatment, two chicken meat sample bags were used; one bag was used for determining the initial population of *C. perfringens* in the sample, at 0 h, and the other was used for determining the final population after anaerobic incubation for 6 h at 37 °C. The population of *C. perfringens* was determined by placing meat samples (10 g) in a stomacher bag containing 90 mL of 0.1% (*w*/*v*) peptone water; aliquots were then serially diluted in 0.1% peptone, plated on BHI agar plates, and incubated anaerobically at 37 °C for 24 h. After incubation, colonies were counted, and final concentrations were determined (CFU/g). All experiments were performed in triplicate using three different spore preparations.

### 2.8. Statistical Analyses

Data were analyzed using analysis of variance (ANOVA) in SAS version 9.3 (SAS Inst. Inc., Cary, NC, USA). Mean values from ANOVA were compared using Duncan’s new multiple range test at a significance level of 0.05. In all figures, the error bars represent the standard deviations.

## 3. Results

### 3.1. Effect of pH on C. perfringens Spore Germination Inhibition by Chitosan and Nisin

**Figure 1:**
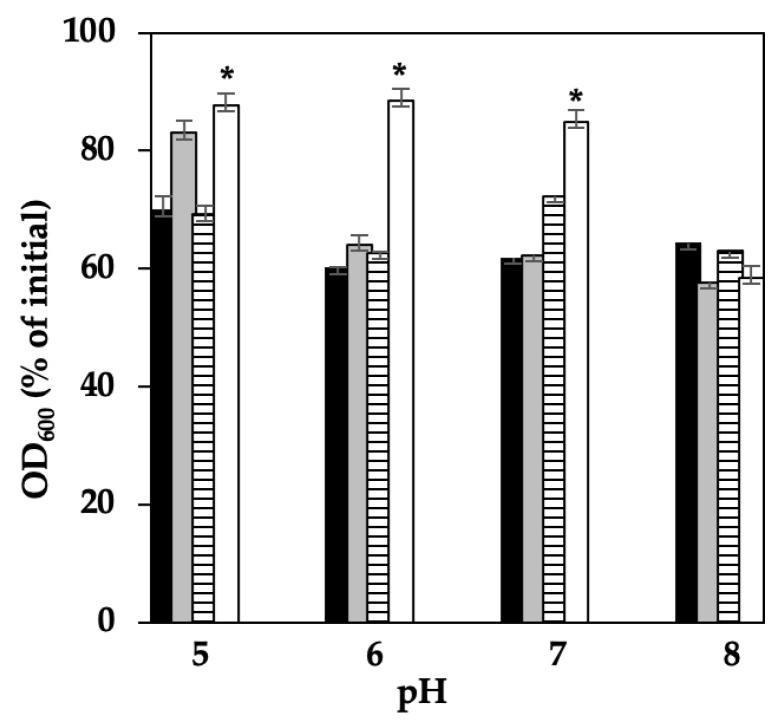
Effect of pH on *C. perfringens* SM101 spore germination. Spores were incubated for 60 min at 37 °C in TGY broth (black bars), TGY + 0.1% chitosan (gray bars), TGY + 0.1% nisin (striped bars), and TGY + 0.1% chitosan + 0.1% nisin (white bars) at different pH values. Germination percentages were calculated based on the decrease in OD_600._ Error bars represent standard deviations from the mean of at least three experiments. * Significant spore germination inhibition (*p* < 0.05).

A previous study from our laboratory showed that the pH of chitosan played a critical role in inhibiting *C. perfringens* SM101 spore germination, with the highest inhibition observed at pH 4.5 [26]. Therefore, we first determined the optimum pH for chitosan–nisin-induced germination inhibition. Spore germination inhibition was determined by measuring the decrease in OD_600_ after incubating *C. perfringens* SM101 spores in TGY medium or TGY supplemented with 0.1% chitosan, 0.1% nisin, or a mixture of 0.1% chitosan and 0.1% nisin at various pHs (pH 5.0–8.0). An approximate 35–40% decrease in OD_600_, compared with the initial OD_600_ value, was observed when SM101 spores were incubated in TGY (pH 5.0–8.0) at 37 °C for 1 h, indicating that SM101 spores germinated well in TGY alone at all these pH values (Figure 1). Similarly, SM101 spores exhibited good germination (approximately 35–40% decrease in OD_600_, compared with the initial OD_600_ value) in TGY supplemented with 0.1% chitosan (pH 6, 7, or 8) or 0.1% nisin (pH 5, 6, or 8). However, SM101 spores germinated poorly in TGY supplemented with 0.1% chitosan (pH 5.0) or 0.1% nisin (pH 7.0), indicating that chitosan and nisin inhibited spore germination at pH 5.0 and pH 7.0, respectively (Figure 1).

Compared with the spore germination values observed in all other treatments, significant (*p* < 0.05) spore germination inhibition was achieved in TGY supplemented with a mixture of chitosan–nisin (0.1% each) within a pH range of 5.0–7.0; treatments at all three pH values led to approximately 10% decrease in OD_600_ compared with the initial value (Figure 1), and no significant differences were observed among the results from these threes pH treatments (*p* > 0.05). Collectively, these results indicate that, although 0.1% chitosan (pH 5.0) or 0.1% nisin (pH 7.0) alone showed a slight inhibition effect on SM101 spore germination, significant inhibition was only observed with chitosan–nisin combination treatments at a pH range of 5.0–7.0.

Given that, in our previous study [20], we observed a significant percentage of germination of SM101 spores in the presence of amino acids at pH 6.0, and that the pH of meat products is close to 6.0, the following experiments were performed at pH 6.0.

### 3.2. C. perfringens Spore Germination Inhibition by a Chitosan–Nisin Combination Treatment at pH 6.0

**Figure 2:**
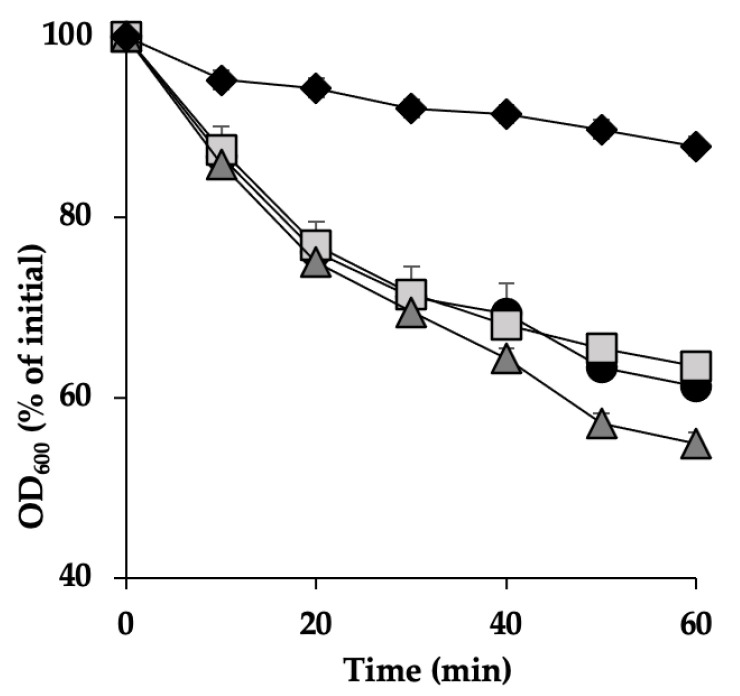
Antimicrobial inhibition of *C. perfringens* SM101 spore germination. Spores were incubated at 37 °C in TGY broth (black circles), TGY with 0.1% chitosan (dark gray triangles), 0.1% nisin (light gray squares), and a combination of both (black diamonds) at pH 6.0. Germination was determined by measuring the decrease in OD_600_ over 60 min. Error bars indicate standard deviations from the mean of three experiments.

We monitored the germination of *C. perfringens* SM101 spores in TGY supplemented with 0.1% chitosan, 0.1% nisin, or a mixture of chitosan and nisin (0.1% each) at pH 6.0 by measuring the decrease in the OD_600_ of each culture at 10 min intervals for up to 60 min. SM101 spores germinated well after incubation in TGY broth alone (pH 6.0); spore germination had already started after 10 min of incubation at 37 °C (Figure 2), and complete germination was achieved after 60 min of incubation (determined by a 50% decrease in OD_600_ compared to the initial value). These results were confirmed using phase-contrast microscopy, which revealed that approximately >95% of the spores became phase-dark, indicating complete spore germination (Figure 2). No significant differences (*p* > 0.05) in germination were observed between SM101 spores incubated in TGY with or without 0.1% chitosan or 0.1% nisin, indicating that neither chemical inhibited spore germination by itself (Figure 2). In contrast, SM101 spore cultures exhibited no significant decrease in OD_600_ (*p* > 0.05; a decrease of approximately 10% of the initial value was observed) after incubation in TGY supplemented with a mixture of 0.1% chitosan and 0.1% nisin for up to 1 h at 37 °C, and >95% of the spores remained phase-bright, indicating significant (*p* < 0.05) inhibition of SM101 spore germination by the combination of these antimicrobials (Figure 2). Notably, the combination of nisin and chitosan at pH 6.0 inhibited spore germination, even though chitosan alone was not active at such pH conditions.

### 3.3. Inhibition of C. perfringens SM101 Spore Germination by Nisin and Chitosan Combination Treatments at Different Concentrations

**Figure 3:**
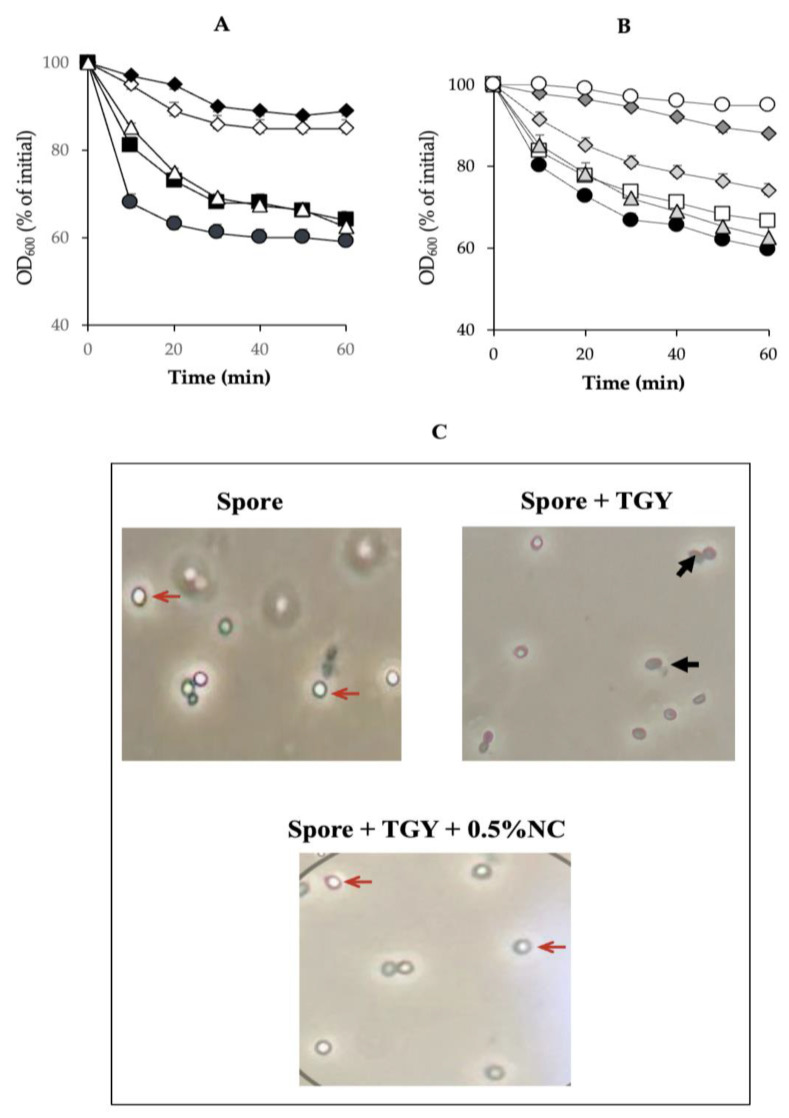
Inhibition of *C. perfringens* SM101 spore germination by chitosan and nisin. SM101 spores were incubated at 37 °C in (**A**) TGY broth (black circles) or TGY with 0.05% chitosan (white triangles), 0.05% nisin (black squares), 0.05% chitosan + 0.05% nisin (white diamonds), or 0.1% chitosan + 0.1% nisin (black diamonds), as well as in (**B**) TGY broth (black circles), TGY with 0.075% chitosan (light gray triangles), 0.075% nisin (white squares), 0.075% chitosan + 0.025% nisin (light gray diamonds), 0.025% chitosan + 0.075% nisin (dark gray diamonds), or 25 mM Tris-HCl (white circles), and OD_600_ was measured at 10 min intervals for 60 min. (**C**) After incubation for 60 min at 37 °C in TGY or TGY with 0.05% nisin + 0.05% chitosan (NC), SM101 spores were visualized under a phase-contrast microscope (100×). Non-germinated spores, thin red arrows; germinated spores, thick black arrows.

To determine the most effective combination of chitosan and nisin for the inhibition of spore germination, we measured the germination of SM101 spores in TGY supplemented with various concentrations of these compounds at pH 6.0. TGY and TGY + 0.1% chitosan–nisin (pH 6.0) treatments were used as positive and negative germination controls, respectively. Our results showed that 0.05% chitosan or 0.05% nisin did not affect SM101 germination, as similar spore germination levels were observed in TGY (positive control) and TGY supplemented (either with 0.05% nisin or 0.05% chitosan) cultures after 60 min of incubation at 37 °C (Figure 3A). However, a combination of chitosan and nisin (0.05% each) significantly inhibited SM101 germination (*p* < 0.01); these cultures showed an OD_600_ decrease of approximately 10%, and >95% of their spores remained phase-bright under phase-contrast microscopy (Figure 3C). In addition, germination inhibition with 0.05% chitosan–nisin was similar to that observed with 0.1% chitosan–nisin (Figure 3A). Further decreasing the concentrations of chitosan and nisin to 0.025% resulted in no inhibition of germination. Although no inhibitory effect was observed with 0.075% chitosan alone, a mixture of 0.025% nisin and 0.075% chitosan inhibited germination (Figure 3B). However, inhibition was highly significant (*p* < 0.01) when a combination of 0.075% nisin and 0.025% chitosan was used (Figure 3B), and SM101 spores germinated very poorly upon incubation under this treatment at 37 °C for 60 min, as OD_600_ decreased by approximately 10% compared with the initial value, and >95% of SM101 spores remained phase-bright (Figure 3B,C). Our results demonstrated that the 0.075% nisin-0.025% chitosan mixture was the most effective combination for inhibiting SM101 spore germination.

### 3.4. Spore Germination Inhibition of Clinically Important Strains Other Than SM101 by a Combination of Chitosan and Nisin

**Figure 4:**
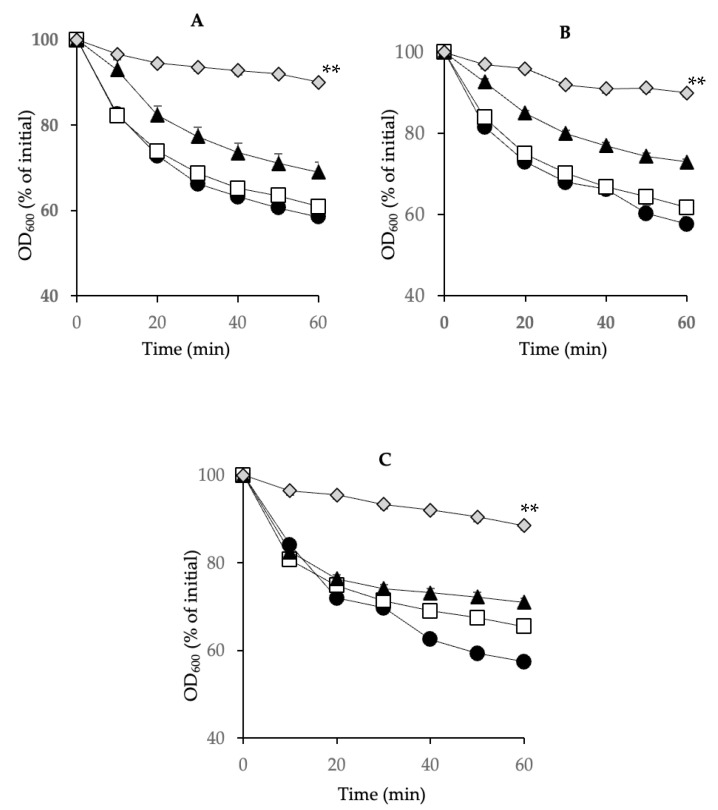
Inhibition of spore germination of FP strains (other than SM101) using a mixture of 0.025% chitosan and 0.075% nisin. Spores of (**A**) NCTC10239, (**B**) NCTC8239, and (**C**) E13 were incubated in TGY broth (black circles) or TGY supplemented with 0.075% nisin (white squares), 0.025% chitosan (black triangles), or 0.025% chitosan + 0.075% nisin (light gray diamonds) at pH 6.0. Germination was assessed by measuring the decrease in OD_600_ every 10 min over 60 min. Error bars indicate standard deviations from the mean of at least three independent experiments. ** Statistically significant inhibition (*p* < 0.01).

To examine whether the identified chitosan–nisin combination was effective against other, if not all, *C. perfringens* FP strains, we performed germination assays using spores of three additional strains, NCTC10239, NCTC8239, and E13, in TGY alone or TGY supplemented with 0.075% nisin, 0.025% chitosan, or a combination of 0.075% nisin-0.025% chitosan. The spores of NCTC10239 and NCTC8239 germinated well in TGY with or without nisin (0.075%), while E13 spores exhibited a slight reduction in germination (non-significant) in TGY with nisin compared to that in TGY alone. These results indicated that 0.075% nisin, by itself, did not inhibit spore germination in any of the tested strains (Figure 4). In addition, 0.025% chitosan supplementation in TGY slightly inhibited germination in all three strains. In contrast, significant (*p* < 0.01) germination inhibition was observed on spores of all the tested strains in TGY supplemented with 0.025% chitosan-0.075% nisin (Figure 4). These results indicate that the 0.025% chitosan-0.075% nisin combination can effectively inhibit germination of spores of most, if not all, *C. perfringens* FP isolates.

### 3.5. Inhibitory Effects of Combined Chitosan and Nisin on C. perfringens Spore Outgrowth

**Figure 5:**
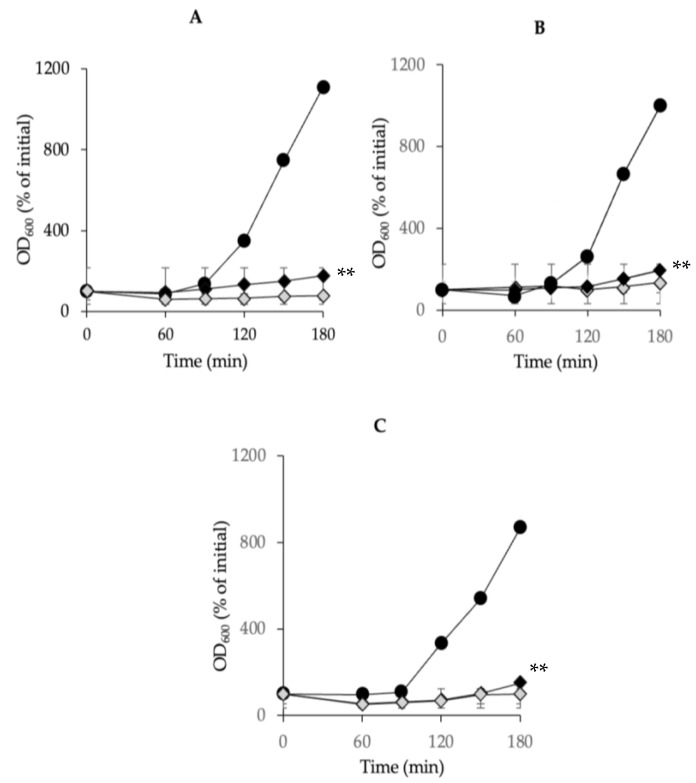
Inhibition of *C. perfringens* spore outgrowth by a mixture of 0.025% chitosan-0.075% nisin. Spores of strains (**A**) SM101, (**B**) NCTC10239, and (**C**) NCTC8239 were incubated in TGY broth (black circles) or TGY supplemented with 0.075% nisin + 0.025% chitosan (light gray diamonds) or 0.01% nisin + 0.01% chitosan (black diamonds) at pH 6.0, and OD_600_ was measured at 30 min intervals for up to 180 min. Spore outgrowth was measured as described in Materials and Methods. Error bars represent the standard deviations from the mean of at least three experiments with three independent spore preparations. ** Significant inhibition of spore outgrowth (*p* < 0.01).

Previous observations in our laboratory suggested that low concentrations of chitosan or nisin could inhibit the outgrowth of *C. perfringens* spores. Therefore, we tested this hypothesis by inoculating *C. perfringens* spores into a rich growth medium. In TGY (pH 6.0), the outgrowth of the spores of the tested strains SM101, NCTC10239, and NCTC8239 was initiated after 90 min of incubation at 37 °C and continued for up to 180 min (Figure 5). However, the presence of a mixture of 0.025% chitosan and 0.075% nisin in the medium significantly inhibited the outgrowth of the spores of the tested strains (*p* < 0.01), as compared with that of the controls in TGY alone; the outgrowth of spores of all tested strains was completely blocked at 180 min of incubation (Figure 5). A similar level of spores’ outgrowth inhibition was observed with a lower concentration combination (0.01% each) of chitosan–nisin (Figure 5). Collectively, our results demonstrated that the combination of chitosan–nisin at very low concentrations effectively arrested the outgrowth of spores in the FP strains.

### 3.6. Effects of a Chitosan and Nisin Combination on the Growth of C. perfringens Vegetative Cells in Laboratory Media

**Figure 6:**
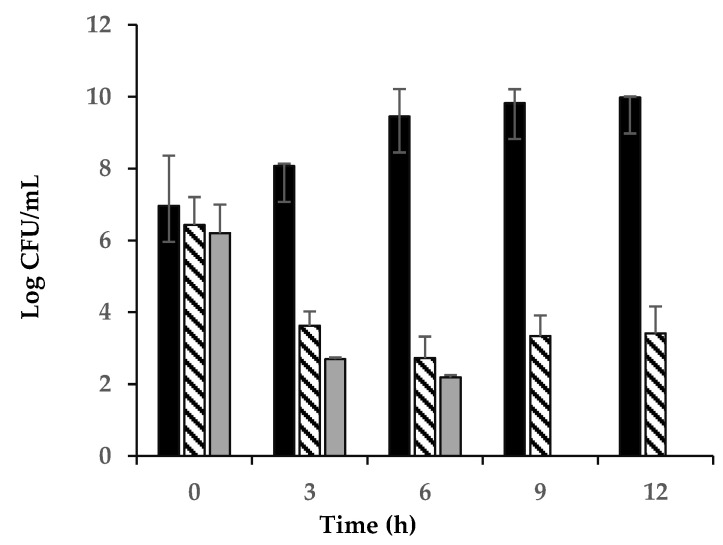
Strain SM101 vegetative cell growth inhibition by different chitosan–nisin concentration combinations. Vegetatively growing cells of strain SM101 were inoculated into TGY broth (black bars) and TGY supplemented with 0.0125% nisin + 0.0125% chitosan (striped bars) or 0.025% nisin + 0.025% chitosan (gray bars), and incubated at 37 °C. Samples were collected at 3 h intervals for up to 12 h, serially diluted, and plated onto BHI agar plates. Viable cell counts (CFU/mL) were determined based on the number of colonies formed after incubation.

We examined the inhibitory effects of the chitosan–nisin combination treatments on the vegetative growth of *C. perfringens* SM101 by incubating SM101 vegetative cells in TGY medium (control) or TGY supplemented with chitosan and nisin at various concentrations at 37 °C for up to 12 h. Serially diluted samples were plated on BHI agar plates and incubated, and the formed colonies were used to estimate viable cell counts (CFU/mL). Our results showed that SM101 cells grew well in TGY alone, increasing the cell population by approximately 2- and 3-log CFU/mL after 3 and 6 h of incubation, respectively, compared to the initial viable cell concentration (Figure 6). However, supplementing the TGY medium with a mixture of 0.025% chitosan-0.075% nisin completely inhibited SM101 vegetative cell survival; therefore, no colonies were detected upon plating 6 h cultures on BHI agar plates. Decreasing the chitosan–nisin level to 0.025% each in TGY also strongly inhibited the survival of vegetative cells, showing approximately 5.0- and 6.5-log CFU/mL reductions in the survival of SM101 cells in the 3 h and 6 h cultures, respectively, compared with that of the control; no colonies were detected after plating aliquots from 9 h and 12 h cultures on BHI agar plates (Figure 6). Further decreasing the concentrations of chitosan–nisin to 0.0125% each in TGY led to approximately 4.0- and 5.5-log CFU/mL reductions in SM101 cell population in 3- and 6 h cultures, respectively, compared to that in the control; no cell count reduction was further observed after 12 h of incubation (Figure 6). Collectively, these results indicate that the combination of chitosan and nisin is bactericidal against *C. perfringens* SM101 cells and that a mixture of 0.025% chitosan-0.025% nisin is sufficient to kill most of these cells.

### 3.7. Together, Chitosan and Nisin Inhibit C. perfringens Spore Germination in Chicken Meat

**Figure 7:**
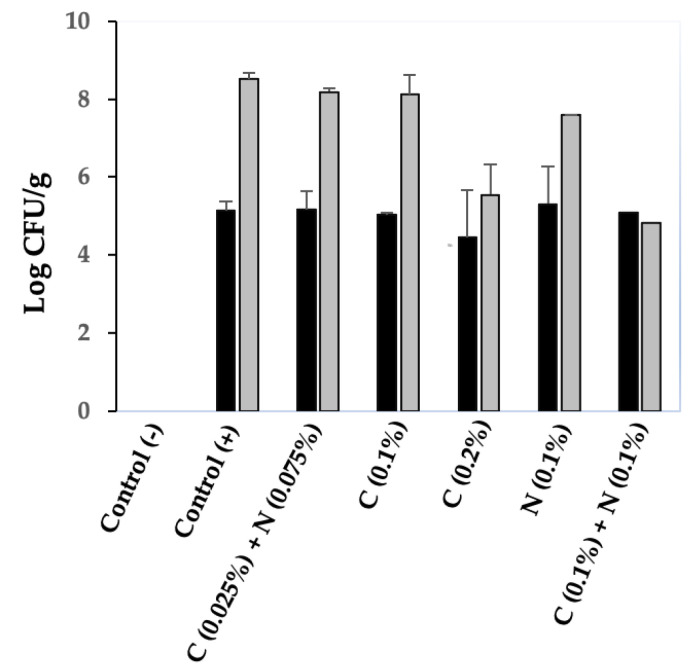
Inhibition of *C. perfringens* 4-strain cocktail spore germination in cooked chicken meat by different concentration combinations of chitosan–nisin. Spores of four different *C. perfringens* FP isolates were inoculated into cooked chicken meat supplemented with different concentrations of chitosan and nisin (as indicated) and incubated anaerobically at 37 °C for 6 h. Viable cell counts (CFU/g) of the survival spores in chicken meat were determined by plating serially diluted samples onto BHI agar plates that were incubated anaerobically at 37 °C for 24 h. Black bars, initial viable cell counts (Log CFU/g) at time 0; gray bars, final viable cell counts (Log CFU/g) in cooked chicken meat after 6 h of incubation; Control (−), spore-free chicken meat; Control (+), chicken meat inoculated with spores; C, chitosan; N, nisin. Error bars represent the standard deviation from the mean of three independent experiments.

A meat model system developed in our laboratory was used to investigate the inhibitory effects of the chitosan–nisin combination on *C. perfringens* spore germination in chicken meat. A *C. perfringens* 4-strain spore cocktail (including spores from strains SM101, NCTC10239, NCTC8239, and E13) was mixed with chicken meat (positive control) or meat supplemented with different concentrations of chitosan, nisin, or both, and incubated anaerobically at 37 °C for 6 h. As expected, *C. perfringens* 4-strain cocktail spores germinated and had favorable growth in cooked chicken meat; a viable cell count increase of >3 log CFU/g was obtained from spore-contaminated cooked chicken meat incubated anaerobically at 37 °C for 6 h versus that at 0 h (Figure 7). Under similar experimental conditions, no growth inhibition of the 4-strain cocktail spores was observed in chicken meat supplemented with a mixture of 0.025% chitosan-0.075% nisin (the most effective concentration combination for the inhibition of spore germination and outgrowth in laboratory medium); an increase of ~3 log CFU/g viable cell count was obtained from chitosan–nisin containing chicken meat upon anaerobic incubation at 37 °C for 6 h versus that at 0 h (Figure 7).

When 0.1% chitosan was added to chicken meat, no inhibitory effect on 4-strain cocktail spore germination was observed; that is, the increase in viable cell counts (CFU/g) was similar to that in the positive control upon anaerobic incubation at 37 °C for 6 h (Figure 7). However, 0.2% chitosan inhibited spore germination in chicken meat, with a viable cell count decrease of ~2 log CFU/g compared to the positive control after anaerobic incubation at 37 °C for 6 h (Figure 7). In contrast, 0.1% nisin in chicken meat resulted in a slight inhibitory effect on spore germination, with a ~1 log CFU/g decrease in viable cell counts compared to that of the positive control (Figure 7). Moreover, the germination of the spores of the 4-strain cocktail in chicken meat was completely blocked when the meat was supplemented with a mixture of 0.1% chitosan-0.1% nisin, and no difference in viable cell counts in chitosan–nisin containing chicken meat after anaerobic incubation at 37 °C for 6 h versus that at 0 h (Figure 7) was observed. Moreover, a >3 log CFU/g decrease in viable cell counts was observed compared with that of the positive control after the 6 h incubation period (Figure 7). These findings suggest that nisin and chitosan are more effective in combination than individually in inhibiting *C. perfringens* spore germination and subsequent proliferation in chicken meat.

## 4. Discussion

Currently, the food industry faces a significant challenge in the development of decontamination strategies, an alternative to thermal processing, that ensures proper eradication of spores without affecting the taste and quality of the treated food products. Such alternative strategies include the application of natural antimicrobial agents in foods to maintain food safety by inhibiting the growth of spoilage and food-borne pathogens. Our previous studies showed that only a high concentration of chitosan (0.2%), but not of nisin (not even at concentrations 4-times higher than those permitted: 0.08%), could effectively inhibit the germination of *C. perfringens* spores in cooked chicken meat. As nisin treatment was more effective when combined with other antimicrobials [27,28,29], in this study, we evaluated the effectiveness of the combination of nisin and chitosan on *C. perfringens* spore germination inhibition.

Our current study has significantly contributed to our understanding of the effectiveness of nisin–chitosan combination on the inhibition of *C. perfringens* spore germination in laboratory medium and chicken meat. First, in combination, nisin and chitosan significantly inhibited spore germination in liquid medium under different pH conditions (from 5.0 to 7.0). While a slight inhibition of spore germination with chitosan at pH 5.0 or nisin at pH 7.0 alone was observed, the significant inhibition observed with the combination of chitosan and nisin suggests a synergistic effect between these two compounds in the inhibition of *C. perfringens* spore germination. Our findings demonstrate that pH 6.0, which also supports optimal spore germination, is a critical point at which inhibition can be effectively achieved using this combination. It can be envisioned that *C. perfringens* spores easily germinate and multiply in meat products (whose pH ranges between 5.5 and 6.5), which are amino acid-rich media, and then the germinated spores may be killed by antimicrobials [13,26].

Second, our study provides clear evidence supporting the fact that nisin and chitosan act synergistically in controlling *C. perfringens* spore germination in liquid medium. We found no germination inhibition in spores exposed to nisin or chitosan alone. However, significant germination inhibition was observed in spores treated with relatively lower concentrations of the nisin–chitosan combination. Hypothetically, chitosan, acts as a permeabilizing agent, affects the integrity of the spore walls/membranes, and thus increases the active diffusion of nisin into the spores [30,31]. Future research to evaluate the effect of nisin on the growth inhibition of chitosan-treated spores or to measure protein/nucleic acid leakage from chitosan-treated spores should clarify the mechanism that underlies germination inhibition. Similar synergistic effects of nisin and other antibacterial agents, including physical, chemical and natural substances against Gram-positive bacteria, have been reported [28,29]. Some examples are: (1) nisin-incorporated blend films of chitosan and carboxymethyl chitosan were more effective against *Listeria monocytogenes* than their pure chitosan counterparts [32]; (2) *Clostridium difficile* vegetative cells were completely inhibited by nisin–lysozyme combinations but not by either compound alone [33]; (3) *C. difficile* spore outgrowth was suppressed by synergistic effects of osmotic activation and nisin [28]. Together with these examples, our findings support the case for synergistic tactics based on nisin and, more significantly, show how applicable they are to the management of *C. perfringens* spores. This implies that the combination of nisin and chitosan may offer a more feasible, low-dose, and efficient way to suppress spore-mediated infections than these therapies alone.

Finally, the most important finding of this study is that, in combination, relatively low concentrations of nisin–chitosan (0.1% each) were sufficient for achieving significant inhibition of spore germination in chicken meat, clearly supporting a synergistic interaction between these antimicrobials. However, these effective nisin–chitosan concentrations (0.1% each) are relatively higher than those (0.075% nisin-0.025% chitosan) obtained for the inhibition of spore germination in laboratory medium. The possible reason for this discrepancy could be attributed to the fact that the efficacy of these antimicrobials in chicken meat is impacted by the intrinsic factors of the meat, including protein, fat, water content, pH, and salt concentration [34]. Because both nisin and chitosan are considered generally recognized as safe (GRAS), the combined use of these chemicals may be a safe and effective means to control *C. perfringens* growth in meat products [29,31]. Therefore, further research on examining the effectiveness of the nisin–chitosan combination against *C. perfringens* spore germination in different types of meats, such as beef, pork, or processed meat products under conditions applied in food industries, may help in developing the effective use of nisin–chitosan combination to control the risk of *C. perfringens-mediated* illnesses.

## 5. Conclusions

One of the most important issues in food safety is managing the germination of *C. perfringens* spores, particularly in thermally processed foods like cooked meat. This study demonstrates the practical potential of combining chitosan and nisin to overcome the limitations of each of these drugs when used separately. In food matrices, the chitosan–nisin synergy demonstrated efficient and encouraging outcomes in lab settings. The development of antibacterial combinations as control methods is supported by these findings as a practical way to improve food preservation without sacrificing quality. Safety and regulatory compliance evaluation, as well as concentration optimization for industrial applications, should be the main areas of future research.

## Data Availability

The original contributions presented in this study are included in the article. Further inquiries can be directed to the corresponding authors.
